# Mediation Effect of Pain on the Relationship between Kinesiophobia and Postural Control: Comparison and Correlations in Individuals with Fibromyalgia Syndrome and Asymptomatic Individuals—A Cross-Sectional Study

**DOI:** 10.3390/life13010175

**Published:** 2023-01-06

**Authors:** Faisal Asiri, Ravi Shankar Reddy, Mastour Saeed Alshahrani, Jaya Shanker Tedla, Snehil Dixit, Adel Alshahrani, Kumar Gular, Abdullah Raizah

**Affiliations:** 1 Department of Medical Rehabilitation Sciences, College of Applied Medical Sciences, King Khalid University, Abha 61471, Saudi Arabia; 2 Physiotherapy Program—Department of Medical Rehabilitation Sciences, College of Applied Medical Sciences, Najran University, Najran 55461, Saudi Arabia; 3 Department Orthopaedics, College of Medicine, King Khalid University, Abha 61471, Saudi Arabia

**Keywords:** kinesiophobia, postural control, fibromyalgia, fear of movement, pain

## Abstract

Background: Individuals with fibromyalgia syndrome (FM) usually present with a fear of movement (kinesiophobia), which causes their symptoms to be maintained and exacerbated. Kinesiophobia can significantly impact postural control; ascertaining their association is crucial in evaluating and managing individuals with FM. This study aims to (1) compare postural control between individuals with FM and asymptomatic individuals, (2) estimate the relationship between kinesiophobia and postural control in individuals with FM, and (3) evaluate whether pain intensity mediates the association between kinesiophobia and postural control in individuals with FM. Methods: This study enrolled 92 individuals (mean age: 51.52 ± 7.7 years) diagnosed with FM and 106 asymptomatic individuals (mean age: 50.47 ± 6.6 years). The examiners estimated the fear of movement and the intensity of pain utilizing the Tampa scale of kinesiophobia (TSK) scores and the visual analogue scale (VAS), respectively. The postural control variables included anteroposterior (A-P) sway in mm, medio-lateral (M-L) sway in mm, and ellipse area in mm^2^. Results: The individuals with FM had impaired postural control compared to the asymptomatic individuals (*p* < 0.001). Kinesiophobia exhibited mild-to-moderate correlations with the postural control variables (nondominant side: A-P sway: r = 0.48, M-L sway: r = 0.49, ellipse area: r = 0.43. Dominant side: A-P sway: r = 0.41, M-L sway: r = 0.33, ellipse area: r = 0.44). The pain intensity significantly mediated the relationship between kinesiophobia and postural control (*p* < 0.001). Conclusion: Kinesiophobia showed a significant positive relationship with postural control. The individuals with FM with higher TSK scores had decreased postural control. Pain intensity mediated the relationship between kinesiophobia and postural control. These factors must be considered when evaluating and formulating treatment strategies for people with FM.

## 1. Introduction

Fibromyalgia syndrome (FM) presents with widespread musculoskeletal complaints, such as pain, fatigue, stiffness, a sense of insufficient sleep, poor physical fitness, and psychological illnesses [1,2]. These factors can significantly impact individuals with FM, leading to increased disability and a decreased quality of life [3,4,5]. Worldwide, FM prevalence is 2–7%, and around 60% of people with fibromyalgia are females [6]. Consequently, it has been a leading cause of rheumatologist visits in the last two decades as a primary condition and comorbidity [5]. Although the pathogenesis of FM is idiopathic, past research indicates that central sensitization processes play significant roles in symptom onset and chronic persistence [7,8].

Kinesiophobia is a term used to define the excessive fear of physical movement in individuals with chronic pain [9,10]. The fear of movement and catastrophic behavior can significantly impact an individual’s ability to perform physical activity [11,12,13,14]. Previous studies have shown a strong relationship between the fear of movement and disability in individuals with chronic musculoskeletal complaints [15]. Individuals experiencing pain for a more extended duration experience fear that engaging in physical activity would exacerbate their conditions, resulting in the avoidance of physical movement or exercise [16]. However, limiting exercise and movement may lead to physical and functional deterioration and depression over time [17]. These components, along with psychological dysfunction, are crucial in prolonging the pain of an acute condition and transforming it into chronic pain [17,18].

The bio-psycho-social framework explains that functional impairment is brought on by a confluence of elements, including pain severity and bio-psychological issues [19]. In this clinical arena, the fear-avoidance model (FAM) explains the relationship between pain and disability and their contribution to developing chronic pain via the psychological process [20]. The fear of movement and catastrophizing thoughts further deteriorate the functional progression of an individual with FM by increasing their disability and decreasing their quality of life [21].

Postural control is essential for maintaining balance, controlling the body’s position in space, and reflecting the body’s sensorimotor function [22]. Different authors have shown increased pain levels, decreased muscle strength, altered activation patterns, impaired proprioception, and increased falls in individuals with FM [14,23]. Previous studies have demonstrated increased sway values compared to asymptomatic individuals [24,25]. Previous studies have shown that elderly individuals experiencing kinesiophobia with chronic pain have an increased disability and a decreased quality of life [26]. Thus, evaluating kinesiophobia and understanding its associations with postural control will aid rehabilitation therapists in formulating treatment guidelines for individuals with FM [27]. Kinesiophobia is a factor that may be associated with FM, and it may magnify postural impairments that need to be evaluated [28].

Persistent pain often occurs among individuals with FM [29]. Different authors have shown a significant relationship between pain severity, the frequency of falls, and balance impairments. Increased pain is associated with increased disability and decreased functional mobility [30]. Previous studies have shown that pain is a significant factor that can cause kinesiophobia and impair postural control. However, how pain influences the impact of kinesiophobia on postural control in individuals with FM is unknown. We employed a mediation analysis using multiple regression to understand the relationship [19].

Although the impact of kinesiophobia has been examined in various musculoskeletal diseases, there is no evidence of how kinesiophobia influences postural control in individuals with FM [31,32]. Therefore, the objectives of this study were to (1) estimate and compare the postural control variables between asymptomatic individuals and individuals with FM, (2) to assess the relationship between kinesiophobia and postural control in individuals with FM, and (3) to assess whether pain intensity mediates the relationship between kinesiophobia and postural control in individuals with FM.

## 2. Materials and Methods

### 2.1. Study Design

This study implemented a cross-sectional study design.

### 2.2. Subjects

This study included 92 individuals diagnosed with FM (mean age of 51.52 ± 7.73 years.) and 106 asymptomatic individuals (mean age: 50.47 ± 6.6 years.). All the subjects participated in this study voluntarily and provided their consent. The study was conducted between 2019 and January to December 2021 in a physical therapy clinic.

### 2.3. Inclusion Criteria

Individuals with FM were included if they (1) met the American College of Rheumatology (ACR) diagnostic criteria for FM (2010 year), as evaluated by a rheumatologist [33]; (2) had a symptom severity (SS) scale score >5 and a widespread pain index (WPI) score >7; (3) had FM symptoms that persisted to a similar extent and for at least three months; and (4) had no other disease or disorder that explained their pain symptoms [33].

### 2.4. Exclusion Criteria

Individuals were excluded if they had (1) fracture, dislocation, or trauma; (2) tumor; (3) any inflammatory joint disease; (4) a history of diabetes mellitus or sensory impairments; and (5) vertigo or vestibular system diseases. Those who were healthy, over 18 years old, and able to comprehend and comply with the instructions given by the examiners were considered asymptomatic participants.

### 2.5. Ethical Considerations

The current study obtained ethical clearance from the ethical board of deanship of scientific research, KKU (ECM#2021-6011), and adhered to the Declaration of Helsinki guidelines.

### 2.6. Outcome Measures

#### 2.6.1. Kinesiophobia

Kinesiophobia is a term that Miller, Kori, and Todd introduced in 1990 at the Ninth Annual Scientific Meeting of the American Pain Society, and it describes a situation where “a patient has an excessive, irrational, and debilitating fear of physical movement and activity resulting from a feeling of vulnerability to painful injury or reinjury” [34]. The Tampa scale of kinesiophobia (TSK) is used to assess the fear of movement of individuals with FM, and it is a reliable and valid scale utilized to estimate the level of kinesiophobia [35]. The original questionnaire was developed to “discriminate between non-excessive fear and phobia among patients with chronic musculoskeletal pain” [34]. The scale has 17 questions. Each question, when answered, is rated by a minimum of 1 (complete disagreement) and a maximum of 4 (complete agreement). The TSK scale has a maximum score of 68 points. If the score exceeds 37, the person likely has kinesiophobia [35,36]. The TSK has been translated and validated in Arabic, and the scale has been found to show excellent reliability (ICC = 0.86) and acceptable internal consistency (α = 0.87) [37].

#### 2.6.2. Pain Intensity

The severity of pain of each participant with FM was assessed using a visual analogue scale (VAS) [38]. The individuals marked a point on a 0 to 10 cm line, representing their pain intensity. The individuals were given an explanation regarding the numbers between 0 and 10 cm. The “0” indicated no pain, and the “10” was the worst possible pain. The justifiable psychometric properties of the VAS scale makes it an ideal tool for assessing pain ratings in different situations [38,39,40].

#### 2.6.3. Postural Control

**Figure 1:**
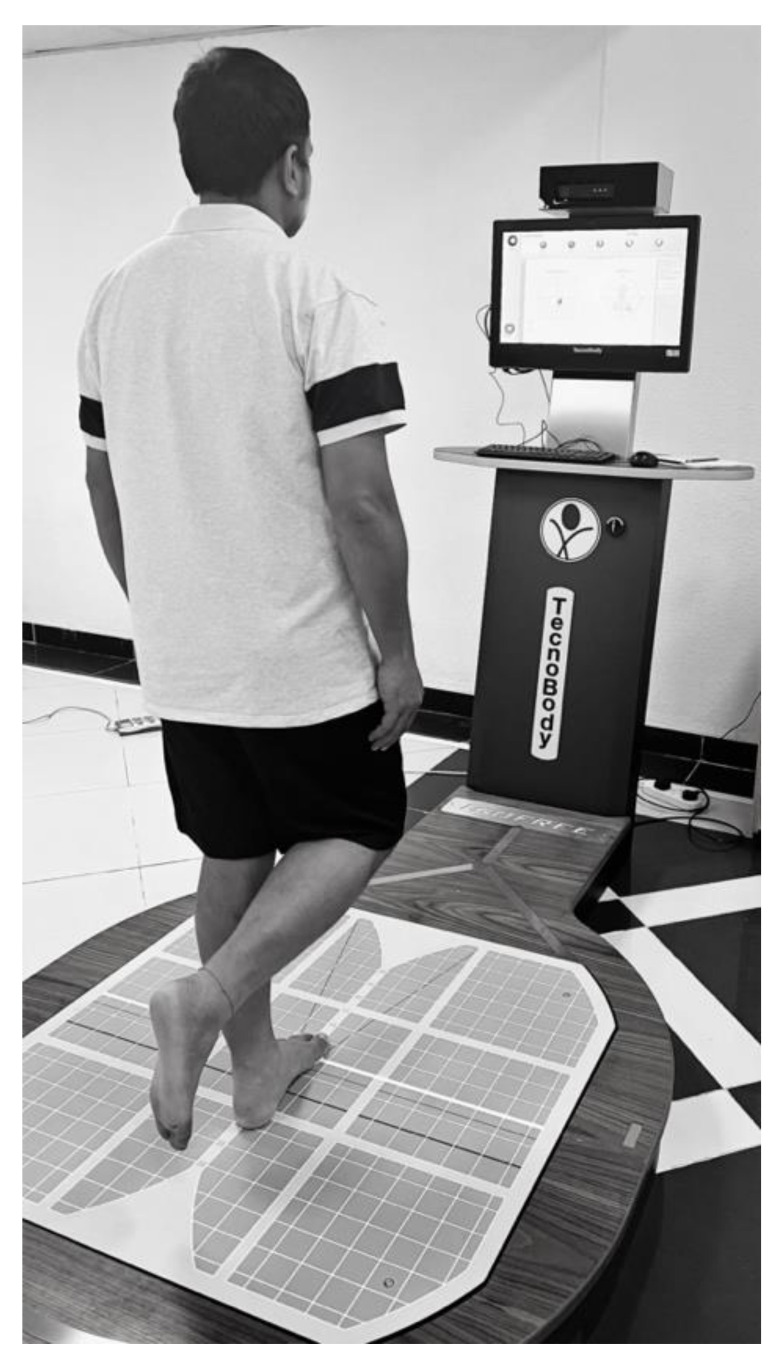
Postural control assessment using a stabilometric platform.

A techno-body stabilometric computerized force platform was used to assess postural control. It consists of four major components: a 3-dimensional camera, a force platform, a touch screen, and software (Figure 1).

The postural control parameters included the velocity of body sway along the anterior–posterior (A-P) and medio-lateral (M-L) directions and ellipse area measurements. The center of pressure is the point of application of forces exchanged between the feet and the ground. The ellipse area is the ellipse that contains the COP trajectory and is measured in mm^2^. All the individuals were given an explanation about the postural control assessments, and an initial trial session was provided before the actual testing. All the tests were carried out in a calm and well-ventilated environment. The stabilometric device was calibrated each day to ensure the accuracy of the results. The participants were asked to stand on the center of the force platform barefooted with one weight-bearing leg (testing leg), while the other (non-testing leg) flexed away from the force platform. During the test, the individuals were instructed to maintain balance while focusing on a specified mark [X] on the system’s monitor for 30 s. Testing was conducted on dominant and nondominant legs, and the leg that was tested first was determined randomly using the chit method. Two trials were conducted for each leg, and the best response of each leg was considered for analyses, whereas the maximum duration of each test was 30 s. The A-P and M-L sway was measured in mm/sec, and the ellipse area was measured in mm^2^.

### 2.7. Statistical Analysis

**Figure 2:**
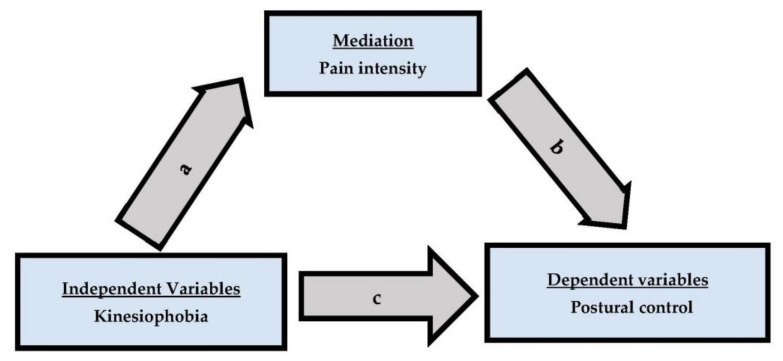
The mediation model includes pain, which is the control variable.

The Shapiro–Wilk test results showed that the study’s data were normally distributed. The Pearson correlation coefficient (r) test was used to assess the correlation between kinesiophobia, pain intensity, and postural control. A mediation analysis was conducted as a four-step process, and it included pain intensity as a mediator (Figure 2). Estimate direct effect (a) between kinesiophobia and pain intensity using bivariate regression. Estimate direct effect (c) between kinesiophobia and postural stability using multiple regression with kinesiophobia and pain intensity as predictors and postural stability as dependent variable. Estimate the direct effect (b) between pain intensity and postural stability using multiple regression with kinesiophobia and pain intensity as predictors and postural stability as dependent variable.

The significance level was determined to be *p* ≤ 0.05. The data from the study were analyzed using IBM SPSS version 24.0 software. G*power statics were employed to estimate the sample size. With an alpha value of 0.05 and a power of 0.80, including the known population mean = 46.1 and the study group mean = 47.5, SD = 4.6), the sample was 85 in each group.

## 3. Results

This study enrolled 92 participants (mean age range: 51.52 ± 7 years) with FM and 106 asymptomatic participants (mean age: 50.47 ± 6.63 years). Table 1 lists the demographic and physical characteristics of the research population. The FMS group was in a range of overweight (BMI = 25.72 ± 4.02) compared to the asymptomatic group.

Postural control was significantly impaired in individuals with FM (*p* < 0.001) compared to asymptomatic individuals (Table 2).

The postural sway and the ellipse area values were larger in the FM group (nondominant: A-P sway = 13.21 ± 4.51, M-L sway = 7.34 ± 2.45, ellipse area = 986.26 ± 152.61; dominant: A-P sway = 9.93 ± 3.83, M-L sway = 6.16 ± 1.83, ellipse area = 966.91 ± 136.10) than in the asymptomatic group (nondominant: A-P sway = 3.33 ± 1.20, M-L sway = 3.90 ± 1.53, ellipse area = 457.47 ± 153.97; dominant: A-P sway = 3.29 ± 1.26, M-L sway = 3.56 ± 1.57, ellipse area = 432.34 ± 154.55). The postural control impairment in the FM group was significant in both the dominant and nondominant sides tested compared to those in the asymptomatic group. The A-P sway, M-L sway, and ellipse area values were larger for the nondominant side tested than for the dominant side tested in both the FM and asymptomatic groups.

**Figure 3:**
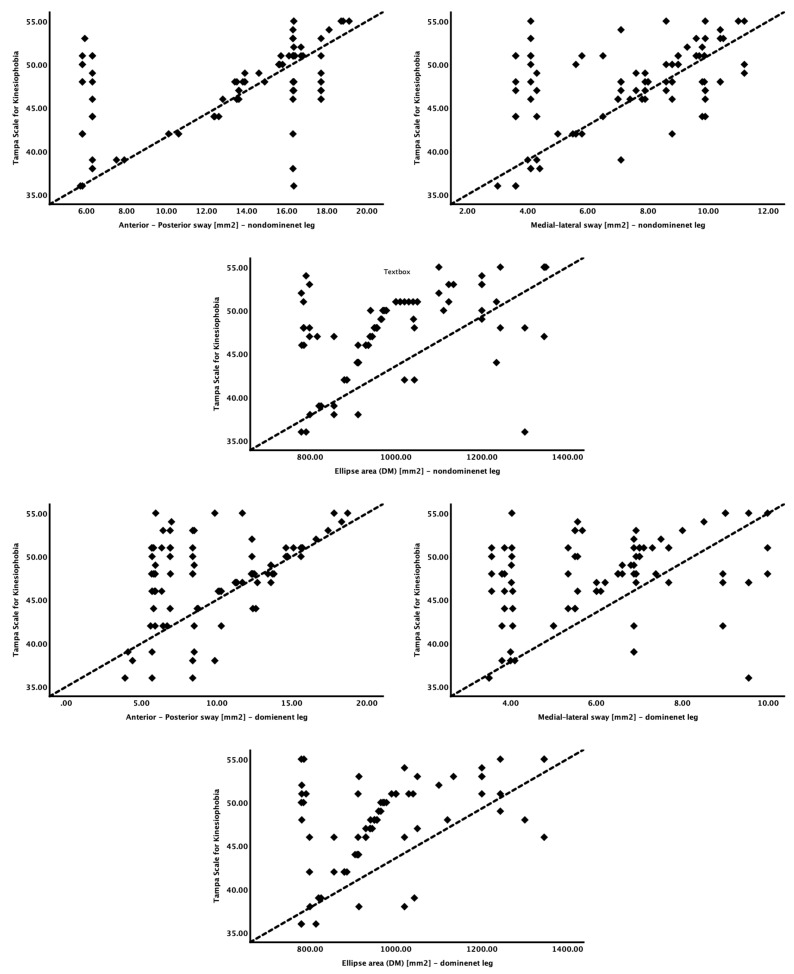
Relationship between kinesiophobia and postural control variables.

Kinesiophobia and its relationships with the explanatory variables are depicted in Table 3 and Figure 3.

Kinesiophobia showed a significant moderate positive correlation with postural control (r = 0.33 to 0.49), with a *p*-value < 0.001. Furthermore, among all the postural control variables, kinesiophobia had the most significant positive correlation with the medial–lateral sway (mm/sec) on the nondominant side (r = 0.49).

The mediation analysis was carried out using pain intensity as a mediator. In step 1, kinesiophobia showed a significant positive association with the postural control variables (A-P sway—nondominant: β = 0.36, *p* =< 0.001; M-L sway—nondominant: = 0.42, *p* =< 0.001; ellipse area—nondominant: β = 13.28, *p* =< 0.001; A-P sway—dominant: β = 0.45, *p* =< 0.001; M-L sway—dominant: =0.83, *p* =< 0.001; ellipse area—dominant: β = 12.13, *p* =< 0.001). Step 2 assessed the direct effect between kinesiophobia and pain intensity and showed a significant association (B = 0.23, SE = 0.01, *p* < 0.001). The results of the mediation analysis (step 3), which evaluated the direct effects of kinesiophobia, pain intensity, and postural control, are summarized in Table 4. There was a statistically significant effect (indirect) when pain intensity was used as a mediator (*p* < 0.05).

## 4. Discussion

This study’s primary aims were to compare postural control between individuals with FM and asymptomatic individuals and to assess the association between kinesiophobia and postural control in individuals with FM. The secondary purpose was to determine whether pain intensity mediates the relationship between kinesiophobia and postural control in individuals with FM. The findings of this study indicate that postural control was impaired in individuals with FM compared to asymptomatic individuals, and kinesiophobia showed a significant positive association with postural control. Furthermore, a mediation analysis showed that pain significantly mediated the relationship between kinesiophobia and postural control.

A limited number of studies have examined the relationship between the fear of movement and postural control in people with FM. The mean TSK score for individuals with FM in this study was 47.5 (SD:4.6), greater than the cutoff limit of 37, indicating that most respondents exhibited kinesiophobia. Consistent with our findings, Russek et al. [41] showed that 72.9 percent of individuals with FM had high levels of kinesiophobia. Few studies have shown a lower percentage of the population to have kinesiophobia. Turk et al. [42] demonstrated that 38.6 percent of the population had TSK score > 37, and van Koulil et al. [43] showed that 40 percent of the sample had kinesiophobia. Disease severity, cultural differences, ethnicity, and the understanding of the disease process may influence kinesiophobia in FM [44].

This study demonstrated impaired postural control in individuals with FM compared to asymptomatic individuals. Muscles and muscle spindles significantly contribute to postural control, and all the trunk and postural muscles should work in coordination to maintain an upright posture with minimal postural deviations [45]. Muscle activation and recruitment patterns may impair postural control in individuals with FM [46]. Musculoskeletal pain and fatigue, primarily in the trunk muscles, can significantly increase postural sway in individuals with FM [47]. As greater rates of fatigue are seen in individuals with FM, this can lead to decreased muscular endurance and diminished muscle force and torque-generating capacity [48]. The postural control in individuals with FM is greatly disturbed by muscle fatigue due to (a) altered recruitment patterns, (b) altered integrated peripheral to central nervous system afferents, and (c) disturbed sensory information [49]. Subjects with greater TSK scores had greater A-P, M-L, and ellipse area displacements in the single-limb stance testing on nondominant and dominant legs. Like our study results, Muthukrishnan et al. [50] showed that individuals with kinesiophobia and constant bodily pain had significantly impaired postural control [50]. Karayannis et al. [51] demonstrated that kinesiophobia and catastrophic behavior could alter postural control in individuals with low back pain [51]. Masood et al. [52] showed that individuals with chronic pain and greater TSK scores had increased A-P sway altering their postural control [52]. These findings demonstrate that patients with FM tend to exhibit postural instability, which is impacted by kinesiophobia, consistent with our hypothesis that postural sway is influenced by the fear of movement.

This study showed that pain intensity significantly mediated the relationship between kinesiophobia and postural control. The fear of pain in many musculoskeletal conditions prolongs the duration of the acute ailment and contributes to its transformation into a chronic condition [53]. Furthermore, increased pain and disuse may result in a loss of muscular tone, deconditioning, and diminished flexibility, which may impair postural control [54]. Pain can affect the neurological system at multiple sites, changing muscle spindle sensitivity and the CNS modulation of proprioceptive afferent inputs [55]. Orfila et al. [56] demonstrated that pain is a significant factor that mediates the relationship between disability and the quality of life in individuals experiencing musculoskeletal pain [56]. Although our investigation revealed the mediating influence of pain on kinesiophobia and postural control, no studies have studied the pathophysiology behind this effect. Additional research is warranted to determine whether pain-relieving interventions can decrease the fear of movement and improve postural control in individuals with CLBP.

### 4.1. Practical Clinical Implications

This study showed that subjects with FM had impaired postural control compared to asymptomatic individuals, and previous studies have shown that the frequency of falls is high in this population [28]. These findings support past research that claimed that patients with FM had increased falls and that demonstrated that kinesiophobia might contribute to balance problems in patients with FM. The outcomes of this study have clinical implications for patients with FM who undergo rehabilitation. In addition, the altered postural sway in individuals with FM was primarily arbitrated due to kinesiophobia, and this factor may be considered when developing treatment strategies to manage patients with FM.

### 4.2. Future Research Implications

We observed that individuals with FM with higher TSK scores had more impaired balance, as assessed using postural stability. There is a direct correlation between impaired balance and an increased frequency of falls. Future studies should consider assessing the direct relationship between the frequency of falls and TSK scores. Moreover, comparing the kinesiophobia levels and their relationship with the frequency of falls between ages and gender will provide vital information for understanding and managing patients with FM.

### 4.3. Limitations of the Study

The subjects were examined using self-reported measures, and their physical activity routine and performances were not analyzed. Additionally, because this trial was not designed with a continuous follow-up, we could not investigate the effect of kinesiophobia on the therapeutic response and success in individuals with FM.

## 5. Conclusions

Kinesiophobia showed a significant relationship with postural control. Pain significantly mediated the relationship between kinesiophobia and postural control in individuals with FM. As kinesiophobia, pain intensity, and postural control are interrelated, these factors should be considered when managing individuals with FM.

## Figures and Tables

**Table 1:** Physical and demographic characteristics of participants.

Variables	Individuals with FM (*n* = 92)	Asymptomatic Individuals (*n* = 106)	*p*-Value
Age (years)	51.52 ± 7.73	50.47 ± 6.63	0.305
Gender (M: F)	39: 53	65: 41	0.008
Height (meters)	1.68 ± 0.10	1.73 ± 0.59	0.001
Weight (kg)	72.20 ± 6.44	69.72 ± 5.23	0.003
BMI (kg/m2)	25.72 ± 4.02	23.28 ± 2.81	0.001
Widespread pain index	13.90 ± 2.50	-	-
Symptom severity score	9.86 ± 1.70	-	-
Kinesiophobia (TSK score)	47.5 ± 4.6	-	-
Pain intensity: VAS (0–10 cm)	5.9 ± 1.3	-	-

FM = fibromyalgia syndrome, BMI = body mass index, VAS = visual analogue scale, SF-36 = short-form 36, TSK = Tampa scale of kinesiophobia.

**Table 2:** Comparison of postural control variables between FM and asymptomatic individuals. (*n* = 92).

Postural Control Variables	Individuals with FM (*n* = 92)	Asymptomatic Individuals (*n* = 106)	f	*p*-Value
Anterior–posterior sway (mm/sec)—nondominant	13.21 ± 4.51	3.33 ± 1.20	470.82	<0.001
Medial–lateral sway (mm/sec)—nondominant	7.34 ± 2.45	3.90 ± 1.53	143.36	<0.001
Ellipse area (mm^2^)—nondominant	986.26 ± 152.61	457.47 ± 153.97	585.71	<0.001
Anterior–posterior sway (mm/sec)—dominant	9.93 ± 3.83	3.29 ± 1.26	281.92	<0.001
Medial–lateral sway (mm/sec)—dominant	6.16 ± 1.83	3.56 ± 1.57	114.86	<0.001
Ellipse area (mm^2^)—dominant	966.91 ± 136.10	432.34 ± 154.55	657.76	<0.001

**Table 3:** Relationship between TSK scores and explanatory variables (*n* = 92).

Explanatory Variables	Kinesiophobia	
	r	*p*-Value
Anterior–posterior sway (mm/sec)—nondominant	0.48	<0.001
Medial–lateral sway (mm/sec)—nondominant	0.49	<0.001
Ellipse area (mm^2^)—nondominant	0.43	<0.001
Anterior–posterior sway (mm/sec)—dominant	0.41	<0.001
Medial–lateral sway (mm/sec)—dominant	0.33	0.001
Ellipse area (mm^2^)—dominant	0.44	<0.001

TSK = Tampa scale of kinesiophobia.

**Table 4:** Direct effects of kinesiophobia, pain intensity, and postural control.

Explanatory Variables	Direct Effect			Indirect Effect		
	B	SE	*p*-Value	B	SE	*p*-Value
Pain intensity × TSK score × Anterior-posterior sway (mm/sec) − nondominant	0.36	0.03	<0.001	0.91	0.03	0.032
Pain intensity × TSK score × Medial–lateral sway (mm/sec) − nondominant	0.42	0.02	<0.001	0.56	0.05	0.023
Pain intensityx TSK score x Ellipse area (mm^2^) − nondominant	13.28	2.13	<0.001	8.35	2.02	0.013
Pain intensity × TSK score × Anterior-posterior sway (mm/sec) − dominant	0.45	0.03	<0.001	0.95	0.07	0.032
Pain intensity × TSK score × Medial–lateral sway (mm/sec) − dominant	0.83	0.04	0.002	0.91	0.16	0.011
Pain intensity × TSK score × Ellipse area (mm^2^) − dominant	12.13	1.82	0.007	9.04	1.81	0.021

TSK = Tampa scale of kinesiophobia.

## Data Availability

All data generated or analyzed during this study are included in Supplementary Information Files.

## Supplementary Materials

The following supporting information can be downloaded at: https://www.mdpi.com/article/10.3390/life13010175/s1, Raw study data of this study are uploaded as a Supplementary File.

## References

[1] Muñoz Ladrón de Guevara C., Reyes del Paso G.A., Fernández Serrano M.J., Montoro C.I. (2022). Fibromyalgia Syndrome and Cognitive Decline: The Role of Body Mass Index and Clinical Symptoms. J. Clin. Med..

[2] Galvez-Sánchez C.M., Duschek S., Del Paso G.A.R. (2019). Psychological impact of fibromyalgia: Current perspectives. Psychol. Res. Behav. Manag..

[3] Galvez-Sánchez C.M., Reyes del Paso G.A. (2020). Diagnostic criteria for fibromyalgia: Critical review and future perspectives. J. Clin. Med..

[4] Alshahrani M.S., Reddy R.S.Y., Tedla J.S. (2020). Effectiveness of Hydro-Galvanic Bath Therapy on Global Health Status, Quality of Life, Depression, and Pain in Individuals with Fibromyalgia–A Quasi-Experimental Study. Phys. Med. Rehabil. Kurortmed..

[5] Alshahrani M.S., Tedla J.S., Reddy R.S., Asiri F. (2020). Effectiveness of Hydrogalvanic Bath on Improving Pain, Disability, and Quality of Life in Individuals with Chronic Nonspecific Neck Pain: A Randomized Controlled Trial. Evid. Based Complement. Altern. Med..

[6] Wolfe F., Brähler E., Hinz A., Häuser W. (2013). Fibromyalgia prevalence, somatic symptom reporting, and the dimensionality of polysymptomatic distress: Results from a survey of the general population. Arthritis Care Res..

[7] Weir P.T., Harlan G.A., Nkoy F.L., Jones S.S., Hegmann K.T., Gren L.H., Lyon J.L. (2006). The incidence of fibromyalgia and its associated comorbidities: A population-based retrospective cohort study based on International Classification of Diseases, 9th Revision codes. JCR J. Clin. Rheumatol..

[8] Alciati A., Atzeni F., Caldirola D., Perna G., Sarzi-Puttini P. (2020). The Co-Morbidity between Bipolar and Panic Disorder in Fibromyalgia Syndrome. J. Clin. Med..

[9] Hapidou E.G., O’Brien M.A., Pierrynowski M.R., de Las Heras E., Patel M., Patla T. (2012). Fear and avoidance of movement in people with chronic pain: Psychometric properties of the 11-Item Tampa Scale for Kinesiophobia (TSK-11). Physiother. Can..

[10] Asiri F., Reddy R.S., Tedla J.S., ALMohiza M.A., Alshahrani M.S., Govindappa S.C., Sangadala D.R. (2021). Kinesiophobia and its correlations with pain, proprioception, and functional performance among individuals with chronic neck pain. PloS ONE.

[11] Larsson C., Ekvall Hansson E., Sundquist K., Jakobsson U. (2016). Impact of pain characteristics and fear-avoidance beliefs on physical activity levels among older adults with chronic pain: A population-based, longitudinal study. BMC Geriatr..

[12] Alahmari K.A., Rengaramanujam K., Reddy R.S., Samuel P.S., Tedla J.S., Kakaraparthi V.N., Ahmad I. (2020). The immediate and short-term effects of dynamic taping on pain, endurance, disability, mobility and kinesiophobia in individuals with chronic non-specific low back pain: A randomized controlled trial. PloS ONE.

[13] Alshahrani M.S., Reddy R.S., Tedla J.S., Asiri F., Alshahrani A. (2022). Association between Kinesiophobia and knee pain intensity, joint position sense, and functional performance in individuals with bilateral knee osteoarthritis. Healthcare.

[14] Alshahrani M.S., Reddy R.S. (2022). Relationship between Kinesiophobia and Ankle Joint Position Sense and Postural Control in Individuals with Chronic Ankle Instability—A Cross-Sectional Study. Int. J. Environ. Res. Public Health.

[15] Wideman T.H., Asmundson G.G., Smeets R.J.M., Zautra A.J., Simmonds M.J., Sullivan M.J., Haythornthwaite J.A., Edwards R.R. (2013). Re-thinking the fear avoidance model: Toward a multi-dimensional framework of pain-related disability. Pain.

[16] Vlaeyen J.W., Linton S.J. (2000). Fear-avoidance and its consequences in chronic musculoskeletal pain: A state of the art. Pain.

[17] Caneiro J., Bunzli S., O’Sullivan P. (2021). Beliefs about the body and pain: The critical role in musculoskeletal pain management. Braz. J. Phys. Ther..

[18] Varallo G., Giusti E.M., Scarpina F., Cattivelli R., Capodaglio P., Castelnuovo G. (2020). The association of kinesiophobia and pain catastrophizing with pain-related disability and pain intensity in obesity and chronic lower-back pain. Brain Sci..

[19] Varallo G., Scarpina F., Giusti E.M., Cattivelli R., Guerrini Usubini A., Capodaglio P., Castelnuovo G. (2021). Does kinesiophobia mediate the relationship between pain intensity and disability in individuals with chronic low-back pain and obesity?. Brain Sci..

[20] Leeuw M., Goossens M.E., Linton S.J., Crombez G., Boersma K., Vlaeyen J.W. (2007). The fear-avoidance model of musculoskeletal pain: Current state of scientific evidence. J. Behav. Med..

[21] Larsson C., Ekvall Hansson E., Sundquist K., Jakobsson U. (2016). Kinesiophobia and its relation to pain characteristics and cognitive affective variables in older adults with chronic pain. BMC Geriatr..

[22] Peterka R.J. (2002). Sensorimotor integration in human postural control. J. Neurophysiol..

[23] Kahraman B.O., Kahraman T., Kalemci O., Sengul Y.S. (2018). Gender differences in postural control in people with nonspecific chronic low back pain. Gait Posture.

[24] Trevisan D.C., Driusso P., Avila M.A., Gramani-Say K., Moreira F.M.A., Parizotto N.A. (2017). Static postural sway of women with and without fibromyalgia syndrome: A cross-sectional study. Clin. Biomech..

[25] Pastor-Mira M.-Á., López-Roig S., Martínez-Zaragoza F., Toribio E., Nardi-Rodríguez A., Peñacoba C. (2021). Motivational Determinants of Objective Physical Activity in Women with Fibromyalgia Who Attended Rehabilitation Settings. J. Clin. Med..

[26] Milenković M., Kocić M., Balov B., Stojanović Z., Savić N., Ivanović S. (2015). Influence of kinesiophobia on activities of daily living of elder institutionalized persons with chronic pain. Prax. Med..

[27] Ishak N.A., Zahari Z., Justine M. (2017). Kinesiophobia, pain, muscle functions, and functional performances among older persons with low back pain. Pain Res. Treat..

[28] Peinado-Rubia A., Osuna-Pérez M.C., Rodríguez-Almagro D., Zagalaz-Anula N., López-Ruiz M.C., Lomas-Vega R. (2020). Impaired balance in patients with fibromyalgia syndrome: Predictors of the impact of this disorder and balance confidence. Int. J. Environ. Res. Public Health.

[29] Fitzcharles M.-A., Cohen S.P., Clauw D.J., Littlejohn G., Usui C., Häuser W. (2021). Nociplastic pain: Towards an understanding of prevalent pain conditions. Lancet.

[30] Peng X., Bao X., Xie Y., Zhang X., Huang J., Liu Y., Cheng M., Liu N., Wang P. (2020). The mediating effect of pain on the association between multimorbidity and disability and impaired physical performance among community-dwelling older adults in southern China. Aging Clin. Exp. Res..

[31] Meeus M., Nijs J. (2007). Central sensitization: A biopsychosocial explanation for chronic widespread pain in patients with fibromyalgia and chronic fatigue syndrome. Clin. Rheumatol..

[32] Ceballos-Laita L., Mingo-Gómez M.T., Estébanez-de-Miguel E., Bueno-Gracia E., Navas-Cámara F.J., Verde-Rello Z., Fernández-Araque A., Jiménez-del-Barrio S. (2021). Does the Addition of Pain Neurophysiology Education to a Therapeutic Exercise Program Improve Physical Function in Women with Fibromyalgia Syndrome? Secondary Analysis of a Randomized Controlled Trial. J. Clin. Med..

[33] Wolfe F., Clauw D.J., Fitzcharles M.A., Goldenberg D.L., Katz R.S., Mease P., Russell A.S., Russell I.J., Winfield J.B., Yunus M.B. (2010). The American College of Rheumatology preliminary diagnostic criteria for fibromyalgia and measurement of symptom severity. Arthritis Care Res..

[34] Luque-Suarez A., Martinez-Calderon J., Falla D. (2019). Role of kinesiophobia on pain, disability and quality of life in people suffering from chronic musculoskeletal pain: A systematic review. Br. J. Sport. Med..

[35] Askary-Ashtiani A., Ebrahimi-Takamejani I., Torkaman G., Amiri M., Mousavi S.J. (2014). Reliability and validity of the persian versions of the fear avoidance beliefs questionnaire and tampa scale of kinesiophobia in patients with neck pain. Spine.

[36] Garrido-Ardila E.M., González-López-Arza M.V., Jiménez-Palomares M., García-Nogales A., Rodríguez-Mansilla J. (2021). Effects of physiotherapy vs. acupuncture in quality of life, pain, stiffness, difficulty to work and depression of women with fibromyalgia: A randomized controlled trial. J. Clin. Med..

[37] Yangui N., Yahia A., Ghroubi S., Elleuch M.H. (2017). Translation and validation of the Tampa Scale of Kinesiophobia Arabic version in chronic low back pain. Ann. Phys. Rehabil. Med..

[38] Mantha S., Thisted R., Foss J., Ellis J.E., Roizen M.F. (1993). A proposal to use confidence intervals for visual analog scale data for pain measurement to determine clinical significance. Anesth. Analg..

[39] Gallagher E.J., Bijur P.E., Latimer C., Silver W. (2002). Reliability and validity of a visual analog scale for acute abdominal pain in the ED. Am. J. Emerg. Med..

[40] Yakut E., Bayar B., Meriç A., Bayar K., Yakut Y. (2003). Reliability and validity of reverse visual analog scale (right to left) in different intensity of pain. Pain Clin..

[41] Russek L., Gardner S., Maguire K., Stevens C., Brown E.Z., Jayawardana V., Mondal S. (2015). A cross-sectional survey assessing sources of movement-related fear among people with fibromyalgia syndrome. Clin. Rheumatol..

[42] Turk D.C., Robinson J.P., Burwinkle T. (2004). Prevalence of fear of pain and activity in patients with fibromyalgia syndrome. J. Pain.

[43] Van Koulil S., Kraaimaat F., van Lankveld W., Van Helmond T., Vedder A., Van Hoorn H., Cats H., Van Riel P., Evers A. (2008). Screening for pain-persistence and pain-avoidance patterns in fibromyalgia. Int. J. Behav. Med..

[44] KOÇYiĞiT B.F., Akaltun M.S. (2020). Kinesiophobia levels in fibromyalgia syndrome and the relationship between pain, disease activity, depression. Arch. Rheumatol..

[45] Borghuis J., Hof A.L., Lemmink K.A. (2008). The importance of sensory-motor control in providing core stability. Sport. Med..

[46] Reddy R.S., Tedla J.S., Dixit S., Raizah A., Al-Otaibi M.L., Gular K., Ahmad I., Sirajudeen M.S. (2022). Cervical Joint Position Sense and Its Correlations with Postural Stability in Subjects with Fibromyalgia Syndrome. Life.

[47] Noguera Carrión C. (2018). Differences between patients with fibromyalgia and chronic low back pain in balance and sensitivity to pain. Univ. Illes Balear..

[48] Estévez-López F., Maestre-Cascales C., Russell D., Alvarez-Gallardo I.C., Rodriguez-Ayllon M., Hughes C.M., Davison G.W., Sanudo B., McVeigh J.G. (2021). Effectiveness of exercise on fatigue and sleep quality in fibromyalgia: A systematic review and meta-analysis of randomized trials. Arch. Phys. Med. Rehabil..

[49] Alvarez M.C., Albuquerque M.L.L., Neiva H.P., Cid L., Rodrigues F., Teixeira D.S., Matos R., Antunes R., Morales-Sánchez V., Monteiro D. (2022). Exploring the Relationship between Fibromyalgia-Related Fatigue, Physical Activity, and Quality of Life. Int. J. Environ. Res. Public Health.

[50] Muthukrishnan R., Shenoy S.D., Jaspal S.S., Nellikunja S., Fernandes S. (2010). The differential effects of core stabilization exercise regime and conventional physiotherapy regime on postural control parameters during perturbation in patients with movement and control impairment chronic low back pain. BMC Sport. Sci. Med. Rehabil..

[51] Karayannis N.V., Smeets R.J., van den Hoorn W., Hodges P.W. (2013). Fear of movement is related to trunk stiffness in low back pain. PloS ONE.

[52] Mazaheri M., Heidari E., Mostamand J., Negahban H., van Dieen J.H. (2014). Competing effects of pain and fear of pain on postural control in low back pain?. Spine.

[53] Blyth F.M., Noguchi N. (2017). Chronic musculoskeletal pain and its impact on older people. Best Pract. Res. Clin. Rheumatol..

[54] Borkum J.M. (2010). Maladaptive cognitions and chronic pain: Epidemiology, neurobiology, and treatment. J. Ration. Emot. Cogn. Behav. Ther..

[55] Henry M., Baudry S. (2019). Age-related changes in leg proprioception: Implications for postural control. J. Neurophysiol..

[56] Orfila F., Ferrer M., Lamarca R., Tebe C., Domingo-Salvany A., Alonso J. (2006). Gender differences in health-related quality of life among the elderly: The role of objective functional capacity and chronic conditions. Soc. Sci. Med..

